# Research on electroconvulsive therapy in India: An overview

**DOI:** 10.4103/0019-5545.69268

**Published:** 2010-01

**Authors:** Bangalore N. Gangadhar, Vivek H. Phutane, Jagadisha Thirthalli

**Affiliations:** Department of Psychiatry, National Institute of Mental Health and Neurosciences (NIMHANS), Bangalore - 560 029, India; 1 Post-Doctoral Associate, Department of Psychiatry, Yale University of School of Medicine, Connecticut Mental Health Centre (CMHC), New Haven, Connecticut, USA

**Keywords:** ECT, Research, Indian

## Abstract

The contribution of researchers from India in the field of electroconvulsive therapy (ECT) has been substantial. Over 250 papers have been published by authors from India in the past five decades on this issue; about half of these have appeared in the Indian Journal of Psychiatry. This article summarizes the papers on ECT research that have appeared in the Journal. A bulk of these articles has focused on establishing the efficacy in different disorders. Considerable numbers of papers describe refinement in the ECT procedure, including anesthetic modification, ECT machine and EEG monitoring. Papers on neurobiology of ECT and long-term follow-up of ECT-treated patients form a minority. Despite the decline in the use of ECT across the globe, papers on ECT have only increased in the recent decades in the Journal.

## INTRODUCTION

### Prologue

**Figure 1:**
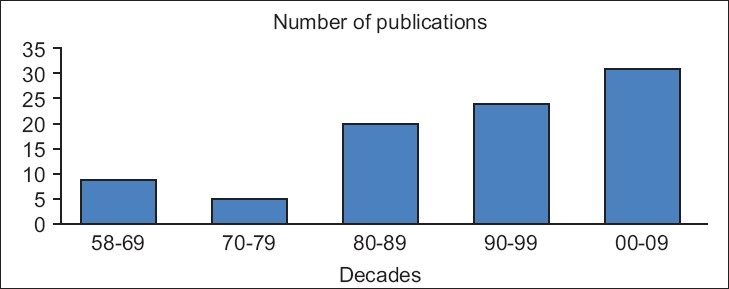
The number of original research papers on electroconvulsive therapy, which appeared in the Indian journal of Psychiatry across five decades

In this essay we attempt to follow research publications on Electro Convulsive Therapy (ECT), in the Indian Journal of Psychiatry, across the last five decades. We searched the database of the Indian Journal of Psychiatry for ECT research articles. We excluded reviews, orations, editorials and letters. More ECT publications from researchers in India have appeared in the recent 3 decades in other indexed journals (PubMed). Clearly, there is a need to examine all available ECT publications from Indian researchers to understand the present status. In the IJP alone, nearly 90 publications have appeared in the last five decades. Figure 1 clearly indicates a steady rise in ECT publications appearing in IJP over these decades; the number in the last decade alone equals the number of all publications in the first three decades. A national workshop on ECT towards the end of the third decade (1989) may have given an impetus to ECT research that explains a sharp increase in the number of ECT publications. Two Tilak Venkoba Rao oration awards of the Indian Psychiatric Society were published on ECT.

### Early trends

Dr. DLN Murthy Rao published the first ever article describing the ectonus method ECT stimulation [Figure 1]. With the development of ECT stimulus machinery, though, this method of stimulation has now become obsolete. Initial reports documented the positive experience of use of ECT in a series of patients of either schizophrenia or manic depressive psychosis. Though ECT was introduced after its success in schizophrenia, the use of ECT in this diagnostic group has now become less frequent. Depression appears to be the most common diagnosis in ECT patients world over; this is not true of India, though. Similarly, researchers recorded the experience with adverse effects of ECT. The observations too were reassuring; the adverse effects were not alarming. Though most ECT services practiced only unmodified ECT for many decades, attempts at studying the role of modifying ECT with some anesthetic medications were published in the initial decade itself. No doubt, this offered a firm foundation for a change in ECT practice towards modified procedures in the later decades (in fact, two of the authors of this paper have not seen unmodified ECT at all).

### Electroconvulsive therapy modification

The current practice includes use of anesthesia and muscle relaxant in modified ECT. Researchers explored different ‘anesthetics’ that include thiopentone, propanadid, etomidate, althesin and diazepam. Propanadid demonstrated some advantages over the more commonly used thiopentone in terms of faster recovery and blunted cardiovascular changes during ECT. The advantages did not perhaps outweigh the cost benefits and practical problems like anesthetists’ experience with propanadid that allowed thiopentone to remain the anesthetic of choice in modified ECT. A survey, conducted early in 1990s, indicates that unmodified ECT was the most prevalent practice in ECT. Muscle relaxation is a serious concern that limits modified ECT practice and calls for a more professional anesthesiologist’s attendance. Yet, as early as 1962, Dr. Bagadia used succinylcholine successfully in modified ECT. A method to assess the extent of muscle relaxation-related seizure modification was also developed as a tool for this area of ECT research, though much later (1999).

The relative merits of retaining the unmodified ECT as regards safety compared to the much-advocated modified ECT have been documented. A more recent study supported use of unmodified ECT if enforcing modified procedure discouraged use of ECT itself. This subject attracted comments on the debate of unmodified versus modified ECT.

### Efficacy of electroconvulsive therapy

Expectedly, schizophrenia was the first condition tested in comparative trials of ECT. ECT added to the concurrent antipsychotic medication benefitted schizophrenia patients. One observation even noted that ECT conferred ‘protection’ against the drug-induced Parkinsonism. In the days of clozapine, a drug recommended for drug-resistant schizophrenias, ECT got a renewed interest. The former drug has feared side-effects of seizures. However, concurrent ECT in clozapine treated patients has not pointed to such concerns, though some precaution has been suggested in such combinations. When used as a first-line of treatment, ECT may just be as good as conventional doses of a standard antidepressant drug though the latter contributed to more adverse effects. In contrast to the well acclaimed dramatic effects of ECT in catatonia, one research report found that lorazepam produced nearly comparable anti-catatonic effects. Yet, ECT is perhaps the first preferred alternative therapy in catatonia when lorazepam is unsuccessful.

### Predictors of electroconvulsive therapy efficacy

Many observations of predictors for response to ECT in depression, noted in standard ECT text books, have been confirmed in independent studies though with some exceptions. The response to the first ECT pointed to a subsequent response in the course for depression. Cardiovascular responses pointed to the potency of the seizure and hence successfully predict the therapeutic efficacy in depression.

### Adverse effects

Ever since the introduction of ECT, the concern over adverse effects prompted research and Indian researchers did not fall behind in this clinic-academic venture. The studies included recording of physiological adverse effects on ECG, intraocular pressure as well as structural brain changes using neuroimaging. ECT passed this screening; sophisticated MRI-based neuroimaging studies failed to detect any brain changes acutely following ECT. Yet case reports of unusual side-effects such as catatonia, pneumothorax, CT-scan evidence of brain changes and even death following ECT have appeared in IJP. Traditional herbs may have the benefit of lowering the memory side-effects of ECT. Dr. Andrade even reviewed the molecular mechanisms of ECT-induced amnesia and possible interventions against such adverse effect. Coexisting medical (even neurological) conditions had usually discouraged clinicians from use of ECT. Observations suggest that ECT can be safely used against psychiatric disorders against the odds of a risk associated with concurrent medical condition. However, this may need additional monitoring mechanisms and expertise.

### Electroconvulsive therapy stimulus

The first report of ectonus type of stimulus has been mentioned earlier. The stimulus laterality has been explored. Bilateral ECT remains the most popularly used as observed in surveys. A model ECT machine of sine wave type of stimulus was assembled and its scientific description appeared in the IJP. It may be remembered that sine wave ECT stimulus is nearly obsolete. A national workshop on ECT, in 1989, suggested designing of indigenous but state-of-the-art ECT machine with pulse stimulus waveform. Very recently, a comparative review of ECT machines of such stimulus output was reported. The author expressed some concerns and a need for a regulatory body for the standards of ECT device

### Monitoring the seizure

Technological advances saw the introduction of EEG monitoring. Initial studies focused on the standardization of EEG monitoring procedures. This included the measurement of ECT seizure duration reliably by noting the change in the seizure morphology. Though EEG monitoring conferred some benefits over mere motor seizure monitoring, the former did not attract ECT practitioners as it carried inherent difficulties, additional expertise, time and cost, to mention a few. Paperless (computer-aided) seizure monitoring may be less cumbersome by avoiding the use of roles of ECT paper.

An alternative physiological measure was also explored, to aid reliable estimation of physiological seizure in ECT, as the behavioral seizure (convulsion) may be missed or is attenuated with modification procedures. Cardiovascular response demonstrated success as a potential alternative; sharp drop in the heart rate following the induction of convulsion marked the end of seizure. Some EEG measures in seizure could be extracted and these had value in predicting the efficacy of ECT.

### Other issues regarding electroconvulsive therapy

Surveys of ECT practice by Indian psychiatrists indicated that we differ from Western practice as regards the frequency of use of modification, choices of conditions prescribed ECT and the ECT stimulus devices. The attitudes of patients and their relatives to ECT have attracted research. The fact that patients as well as their relatives have a favorable attitude towards ECT may also have allowed more patients to receive and benefit from ECT; and hence, contribute to the bulk of ECT research publications.

### Anything left?

The mechanisms of action of ECT have been poorly explored as seen in the published ECT research. Some animal research has appeared pointing to dopamine receptor changes following electroconvulsive shocks (a model of clinical ECT). Among human studies, the publications included comparison of DST results and platelet 5-HT uptake between ECT and imipramine treated patients. Have researchers from India published more on this subject in other indexed journals? This area merits more investigation with the use of modern technology such as magnetic resonance spectroscopy.

## NOTE ON THE SEARCH

A search was conducted in all volumes of Indian Journal of Psychiatry from 1959 till 2009. The search was carried out using the following key words: Electroconvulsive therapy, electroconvulsive, ECT, E.C.T. The study has included all studies related to humans, original articles, case series, case reports and excluded animal studies, review articles, editorials and letters that are not data-based, as they are opinions of various authors and not the real clinical data.
